# Comparative performance information plays no role in the referral behaviour of GPs

**DOI:** 10.1186/1471-2296-15-146

**Published:** 2014-08-27

**Authors:** Nicole ABM Ketelaar, Marjan J Faber, Glyn Elwyn, Gert P Westert, Jozé C Braspenning

**Affiliations:** Radboud university medical center, Scientific Institute for Quality of Healthcare 114, P.O. Box 9101, 6500 HB Nijmegen, The Netherlands; Radboud university medical center, Scientific Institute for Quality of Healthcare, Nijmegen, The Netherlands; The Dartmouth Health Care Delivery Science Center and The Dartmouth Institute for Health Policy and Clinical Practice, Dartmouth College, Hanover, N.H. USA

**Keywords:** Primary care, Doctor-patient relationship, Access to care, Performance information, Quality of care, Qualitative research, Quantitative research, Mixed methods

## Abstract

**Background:**

Comparative performance information (CPI) about the quality of hospital care is information used to identify high-quality hospitals and providers. As the gatekeeper to secondary care, the general practitioner (GP) can use CPI to reflect on the pros and cons of the available options with the patient and choose a provider best fitted to the patient’s needs. We investigated how GPs view their role in using CPI to choose providers and support patients.

**Method:**

We used a mixed-method, sequential, exploratory design to conduct explorative interviews with 15 GPs about their referral routines, methods of referral consideration, patient involvement, and the role of CPI. Then we quantified the qualitative results by sending a survey questionnaire to 81 GPs affiliated with a representative national research network.

**Results:**

Seventy GPs (86% response rate) filled out the questionnaire. Most GPs did not know where to find CPI (87%) and had never searched for it (94%). The GPs reported that they were not motivated to use CPI due to doubts about its role as support information, uncertainty about the effect of using CPI, lack of faith in better outcomes, and uncertainty about CPI content and validity. Nonetheless, most GPs believed that patients would like to be informed about quality-of-care differences (62%), and about half the GPs discussed quality-of-care differences with their patients (46%), though these discussions were not based on CPI.

**Conclusion:**

Decisions about referrals to hospital care are not based on CPI exchanges during GP consultations. As a gatekeeper, the GP is in a good position to guide patients through the enormous amount of quality information that is available. Nevertheless, it is unclear how and whether the GP’s role in using information about quality of care in the referral process can grow, as patients hardly ever initiate a discussion based on CPI, though they seem to be increasingly more critical about differences in quality of care. Future research should address the conditions needed to support GPs’ ability and willingness to use CPI to guide their patients in the referral process.

## Background

As comparative performance information (CPI) about healthcare service, patient experiences, and quality of clinical care becomes increasingly available, questions about its use arise, as do questions about general practitioner (GP) views of CPI at the time of referral. In healthcare systems where the GPs are the gatekeepers of secondary care, which they are in the Netherlands and the UK, GPs refer their patients to specialists for further examination, diagnosis, or treatment. In doing so, they play an important intermediary role between patient and hospital [1–3]. International studies have shown that the referral is traditionally affected by previous experiences with specialists, perceptions of specialists’ interactions with patients, office location, specialists’ medical skills, and patient preferences [4–7]. These traditional considerations are all understandable. The current focus on the patient’s choice of healthcare provider, with CPI for identifying the quality of provider performance [8, 9], calls for GPs to reflect anew on the current referral process.

The patient’s involvement in choosing a healthcare provider in the Netherlands has been encouraged since regulated competition was introduced during the 2006 healthcare system reform. Publicly available CPI introduced to encourage this competition, contains information about the performance, quality of care and is available for various providers [10], also patient experiences plays an increasingly important role. The information covers items at the hospital level (patient volumes, inspection scores defined by the Dutch Health Care Inspectorate, which includes specific conditions such as waiting lists, treatment volumes, treatment methods, methods of anaesthesia, number of specialists treating a given condition, and patient experiences [11].

The CPI can make an impact when patients select providers of high-quality care on the basis of this kind of information. However, patients hardly use such information for selectively choosing a provider [12, 13]. Bringing such a choice into practice is a difficult and complex task for patients; e.g. they do not know how to set their own values [14, 15]. The CPI can be difficult to interpret, especially when it contains conflicting criteria, shows multiplicity formats, or the presentation makes it difficult to understand [14, 16, 17]. Given the lack of CPI usage among patients for selectively choosing a provider, we are looking for ways to provide additional support for the patients. Schlesinger and colleagues advise providing advocates who can help patients with their choices of hospital and who can act on their behalf if they have difficulties putting their choices into practice [18]. From the patient’s perspective, this advocate could be the GP. Patients do not seem to search for CPI themselves, but they do ask for advice when choosing a healthcare provider. The GP is an important advisor for about half the Dutch patients [19] because patients consider their GP to be a reliable source of quality information [20]. Dutch research confirms that GPs have significant influence in directing patients: 68% of the patients who searched for information to select a hospital noted that they based their final decision on GP advice [21].

Several studies have revealed how providers respond to performance information [22–25]. A 1996 study among cardiologists and cardiac surgeons shows that the publication of report cards for grafts bypassing the coronary artery has little credibility and therefore little influence on referral recommendations [25]. A mixed group of physicians described several issues that made them sceptical of the data and concerned about using the information with patients [23]. Further, it appears that quality-of-care data have little impact on referral decisions [22]. This paper addresses the following research questions:

- Can the GP be a choice-supporting advocate for helping patients use comparative performance information?
- What are the current referral considerations?
- What is the GP’s perception of patient involvement in referral decisions and the use of comparative performance information?
- What factors constrain GPs in using comparative performance information in the referral process?

We conducted explorative interviews to review their referral routines in which we included the current considerations, patient involvement, and the role of CPI in referral decisions. Using the results of these interviews, we designed and conducted a quantitative survey with a representative sample of Dutch GPs.

## Methods

### Design

We used a mixed-method, sequential, exploratory design [26]. In this design, the qualitative element is considered first for exploring the research area, then the quantitative element is used to extend and quantify the qualitative results [26]. The methods are integrated in three ways. First we focused on *building,* while the interview results are used in the data collection to build the survey [27]. The second way was *merging*: we used both databases for analysis and comparison. Thirdly, we transformed qualitative data to quantitative data, then integrated the results with illustrative quotes [28]. A small part of the qualitative data was not transformed in the survey, though it will be used in the results.

### Participants

For the explorative interviews, we recruited GPs from the Nijmegen University Network of General Practitioners and from a network of innovative primary care projects financed by a Dutch healthcare insurance company. The resulting survey questionnaire, designed to quantify the issues raised in these interviews, was distributed among a sample of 81 primary care practices affiliated with a representative national network of general practices. Participation was voluntary, and no incentives were offered. The study has been carried out in the Netherlands in accordance with the applicable rules concerning the review of research ethics committees and informed consent.

### Explorative interviews

**Figure 1:**
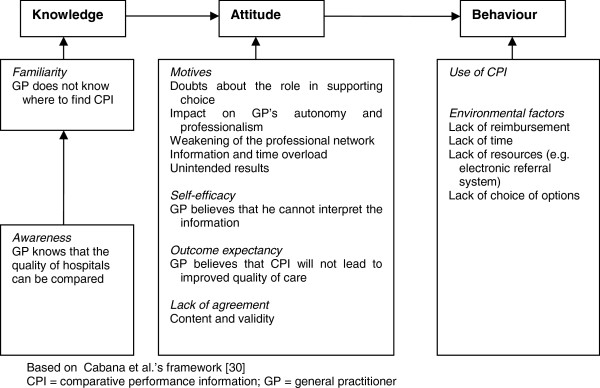
**Barriers to the use of comparative performance information by GPs in relation to their referral behaviour.**

We conducted explorative interviews with 15 GPs that focussed on referral routines related to three main issues, namely (1) referral considerations (which and why), (2) patient involvement in the referral process in general, and (3) the role of comparative performance information during referral in terms of knowledge about CPI, attitudes towards it, and actual usage behaviour. The first author (NK) interviewed all the GPs. The interviews lasted from 30 to 45 minutes. The interviews were audio-recorded and transcribed verbatim. Two researchers (NK and MF) analysed the transcripts. First, they read the interviews to obtain a comprehensive impression of the material. Second, the data were extensively and inductively coded. Indexing the data created a large number of codes that were repeatedly refined and reduced in several rounds [29]. Third, with regard to the role of comparative performance information, a framework analysis was used to approach the data deductively. Cabana and colleagues developed the general guidelines for this framework for improvement [30]. The use of CPI and the use of professional guidelines differ, though there is a resemblance in the way they both could be implemented. The successive steps helped us analyse the interviews, and we used them as a guide to present the results in the section ‘Use of comparative performance information’. Figure 1 shows the findings for this part of the interviews. We used the analysis software Atlas.ti.5.2 to facilitate the coding process [31].

### Survey

We built on the issues that arose in the interviews and transformed them into a survey questionnaire. The CPI-related questions were added to the annual survey of the Dutch National Information Network of General Practice (LINH). The LINH has a nationally representative database maintaining longitudinal data derived from patients’ electronic medical records about consultations, morbidity, drug prescriptions, and referrals [32]. The LINH consists of 81 general practices with approximately 335,000 patients. The data were collected between September and December 2012. The survey focused on GP considerations in the current referral process, GPs’ views towards patient involvement, GPs’ current experiences with patient involvement, and the role of comparative performance information in referral decisions. The seven items in GP considerations in current referrals were patient preferences, experiences of other patients, waiting lists, quality of care, specific treatment or techniques, patient travelling distance, and personal contact with a specialist. The question *‘to what extent did the GP consider these items in the decision to refer a patient’* was to be answered on a five-point Likert scale ranging from 1 (never) to 5 (always).

Five points for patient involvement were formulated: (1) *to what extent did GPs agree with patients’ needs for information about quality differences*, (2) *how often did patients refer to* comparative performance information *during consultations,* (3) what were *GPs’ evaluations of patients’ use of quality information about hospitals,* (4) how did *GPs perceive the ability of patients to decide on a hospital themselves*, and (5) *what about the GPs’ view that the use of CPI is the patient’s own responsibility?* A five-point rating scale ranging from 1 (totally disagree) to 5 (totally agree) was used.

The main part was about the role of comparative performance information in referral decisions (21 items). The findings of Figure 1 about knowledge, attitude, and behaviour were listed in the items. The GPs were asked if they knew where to find information about quality of care, and whether they searched for CPI (both dichotomous variables). Ten statements about comparative performance information, containing the elements of attitude and behaviour, were developed. They included elements of quality-of-care differences between hospitals, GP use of comparative performance information to select a hospital, the GPs’ role and responsibility regarding the use of comparative performance information, effects of CPI on the continuity of care, time management regarding comparative performance information, and GP views of the use of CPI in the next 5 years. For the descriptive analyses, the ratings of these items were transformed into the percentages of GPs who agreed (rating of 4 or 5 on the Likert scale). Finally, GPs were asked to express their opinions about the currently available CPI with respect to credibility, transparency about how information was gained, contradictory sources, user friendliness, is it up to date, comprehensibility, the ability of CPI to show quality of care differences, connection to patients’ wishes, and the information content. A five-point scale ranging from 1 (totally disagree) to 5 (totally agree) was used; ‘Do not know’ was a separate option in a separate column. We gathered general practice characteristics about the locality of the practice (urbanization), number of patients in the practice, and the practice type (single or group practice). Background information about the GP included gender and the number of days a week the GP was available in the practice (part time or full time). The data are presented in terms of means (s.d.) or percentages (%).

## Results

The response rate of the survey was 86%. Table 1 presents the characteristics.

**Table 1:** **Characteristics of the 70 participating general practitioners and their practices**

	Number	%
**GP characteristics**		
Male	55	80
Full-time GP	66	52
**Practice characteristics**		
Single-handed practice	38	54
Duo practice	12	17
Group practice	12	17
Healthcare centre	8	12
**Urbanization level**		
Very high	20	29
High	11	16
Moderate	14	20
Low	14	20
Rural	11	16

### Current referral considerations

In the interviews, GPs spoke in great detail about their preference for using their own prior experiences and personal contacts with specialists or hospitals when considering their referral. Personal contacts were important to the GPs because they provide an opportunity to ask medical questions and to estimate colleagues’ interactions with patients. In contrast, the surveyed GPs stated that, in deciding about a referral, they primarily considered patients’ preferences for a hospital or a provider, then the quality of care, and then the distance from the patient’s home (Table 2).

**Table 2:** **The importance of factors in the referral process for selecting a hospital or a specialist**

	Mean (s.d.) of the 70 responses
Patients’ preferences for a hospital or provider	4.3 (0.7)
Quality of care	3.8 (1.0)
Patient’s travel distance to a hospital or provider	3.8 (0.9)
GP’s personal contact with a specialist	3.6 (0.9)
Waiting list	3.5 (0.8)
Specific treatment or techniques	3.5 (0.8)
Experiences of other patients	3.4 (1.0)

The responses were given on a five-point Likert scale, with ‘5’ representing ‘always taken into consideration’, and ‘1’ as ‘never taken into consideration’.

Standard deviation: s.d.

*‘I mainly refer on personal grounds and experiences. This might be a really bad thing to do. Still, I think it works this way.’ (N 1)*

### Patient involvement

During the interviews, the GPs said that they always started by asking what the patient wanted, and they repeatedly highlighted the fact that when selecting a healthcare provider, patients valued other choice attributes than those reflected in CPI. The GPs also noted that comparing providers and making a rational trade-off based on CPI are difficult tasks for patients.

*‘Familiarity with a hospital, distance, and knowing where to go: these are much stronger arguments for the patient than quality of care.’ (N 10)*

*‘The mortality rates in hospital A are better than in hospital B, but A is a generic hospital while B is a top clinical one with an intensive care unit. For that reason, there is a greater chance that people will die in hospital B. The mortality rates show you the data, but you need to interpret them with the background information in your head. As a doctor I can do that, but patients?’ (N 6)*

There was a high level of agreement between the surveyed GPs about the importance of patients making their own hospital choice and needing to be informed about differences in quality of care (for both items, M = 4.0; s.d. = 0.8). There was less agreement about the statements that quality information about hospitals has added value for patients (M = 3.0; s.d. = 0.8) and that the use of CPI is a patient’s own responsibility (M = 3.0; s.d. = 0.7). There was little GP agreement about how often patients refer to CPI during consultations (M = 2.3; s.d. = 0.9). Approximately half the GPs (47%) agreed that they were ‘sceptical about patient use of CPI’ (M = 3.0; s.d. = 0.9).

### Use of CPI

#### Knowledge

Most GPs (83%) reported that they did not know where to find performance information to compare their regional hospitals.

#### Attitude: motives

The GPs varied widely in their attitude towards CPI. We distinguished four motives that shaped GPs’ negative attitudes towards the use of CPI. First, the GPs want the best care for their patients, and they doubt the role that CPI plays in supporting referral choices. They suggested that they would like to have a ‘tailored’ referral process because the extent of patient involvement in choosing a hospital varies. About half of the surveyed GPs (47%) agreed that they have a task in supporting patients’ hospital choice based on CPI. They recognized CPI as a type of information that can facilitate patient involvement in choosing a hospital.

*‘Patients want to steer their decisions.... When they are old and weak they say, ‘Put me in the back seat and drive me to the nearest hospital.’ Other patients say, ‘Sit next to me and tell me how, but I am the one who’s driving.’ And there is a group of patients who ask, ‘Where is the highway to the best specialist in this area?’ (N 14)*

A second motive reported in the interviews was that the use of CPI interferes with the GP’s professional role. The GPs felt responsible for an optimal referral and emphasized their role as coordinators. They expected patients to rely on their referral advice. Therefore, they needed to adjust to the idea that patients can now propose alternatives. They were afraid that patients could decide to go to hospitals outside their professional network as a result of CPI. This could increase the number of medical specialists GPs have to deal with and thereby impede communication since it is easier to talk to someone you know. The GPs also greatly valued patients’ anecdotal reports, so if the size of the professional network were to increase, these patient reports would become more difficult to interpret.

*‘When people came up with propositions to go elsewhere, I was unprepared. The way I was educated to deal with my profession as a GP - it was all about personal contact with specialists and providers, not about the arguments of performance information gathered elsewhere or waiting lists.’ (N 13)*

As a third motive in the interviews, the GPs expressed concerns regarding unintended consequences of using CPI during the referral process. This might limit the accessibility of certain types of care for those patients who could not easily choose to go elsewhere. Almost half the GPs surveyed (46%) agreed that referral based on CPI could increase fragmentation of care and threaten the continuity of care.

*‘You can be treated in many hospitals for all kinds of things, but you need to have some sort of continuity in your treatment, which often means you end up in the same hospital.’ (N 15)*

Fourth, during the interviews several GPs said that it would take too much time to remain up to date about the CPI for multiple conditions and for a range of patient groups, even though only a minority (23%) of surveyed GPs agreed that ‘the use of CPI takes too much time’.

#### Attitude: outcome expectancy

The GPs had doubts about the outcome expectancy for CPI. In the interviews, some questioned whether using CPI would lead to an improvement in quality of care. The surveyed GPs were also divided in their opinions about the differences in quality of care in hospitals: 66% disagreed with the statement that the quality of care varies greatly between hospitals.

#### Attitude: self-efficacy

During the interviews, some GPs said they were unsure whether they could interpret CPI information and make a trade-off based on all the available information. The results in Table 3 show that it is difficult for GPs to interpret CPI information.

**Table 3:** **General practitioners’ level of agreement about statements concerning outcome expectancy, content, and validity of comparative performance information**

Statements	Totally agree/disagree	Do not know
	Mean (s.d.)	%
CPI is not transparent about how information is determined	3.6 (0.7)	18
CPI is not clear because of contradictory sources	3.6 (0.7)	20
CPI is not credible	3.2 (0.7)	20
CPI is not in line with patients’ wishes	3.2 (0.6)	27
CPI is difficult for patients to understand	3.3 (0.7)	21
CPI is not specific enough	3.3 (0.7)	21
CPI is not user friendly	3.3 (0.7)	23
CPI gives the wrong choice attributes	3.2 (0.6)	20
CPI is not up to date	3.1 (0.6)	28
CPI has no ability to show differences in quality of care	3.0 (0.6)	20

The 68 responses were given on a five-point Likert scale, with ‘5’ representing ‘Totally agree’ and ‘1’ representing ‘Totally disagree’. ‘Do not know’, was a separate sixth answer possibility.

Standard deviation: s.d.

*‘It is really difficult to assess the quality of care provided by my colleagues, and they are in the same building! Not to mention colleagues elsewhere or specialists in the hospital. It is almost impossible to make good judgements about that.’ (N 8)*

#### Lack of agreement with content and validity elements

The GPs had their doubts regarding both the content itself and the validity of CPI (Table 3). A fairly large proportion of the GPs said ‘I do not know’ when they were asked about various CPI elements.

In the interviews, some GPs mentioned conflicts of interest. They felt that the sources on which CPI is based should include disclaimers about various aspects of data collection and validity, and that the sources should declare any conflicts of interest.

*‘I do not know about using CPI for referral decisions, but I get the feeling that I’m promoting a particular hospital.’ (N 9)*

#### Behaviour

Most of the GPs (94%) declared that they had never searched for hospital performance information in their region; however, 12% reported that they had used CPI for selecting a hospital. Further, approximately half the GPs agreed that they had discussed quality-of-care differences between hospitals with their patients (M = 3.2; s.d. = 0.8). The GPs were undecided regarding the expectation that CPI will becomes a part of their referral advice within 5 years – only a minority agreed with this statement (M = 3.0; s.d. = 0.8).

*Interviewer: ‘Do you believe that patients will make more informed choices in the future?’*

*GP: ‘Frankly? No, I do not think so.’ (N 4)*

#### Environmental factors

A lack of reimbursement, time to search for CPI, the small number of hospitals to choose from, and not having an electronic referral system containing CPI were mentioned in the interviews. About half the surveyed GPs (48%) noted that they lacked an electronic system to help them use CPI in the referral process.

## Discussion

Can the GP be a choice-supportive advocate for patients to overcome patients’ lack of CPI usage? Our study shows that we cannot expect that a GP can play an advocate’s role in the use of CPI. Currently, GP considerations at the point of referral are patient preferences, quality of care, and travel distance, and there is no role for CPI as an additional source. The GPs feel that patients should become more aware of quality differences in general. They do not believe that current CPI has any added value for patients. The GPs rarely see patients initiating a discussion about CPI during consultations, and most are sceptical about the ability of patients to use CPI. The GPs’ own use of CPI is hindered by several barriers, including indecisiveness about their role in supporting patients’ choices and their task in addressing CPI during consultations.

### Comparison with existing literature

The healthcare reforms in north-western European countries have been designed to encourage a greater role for patients in choosing a provider and to spur providers on to support this choice. The purpose of this design is to increase the competition between providers for the benefit of the patients [33, 34]. Our results show that current practice does not yet support the concept of GPs acting as agents of patient choice and users of CPI. The GPs in our study rarely had patients who mentioned CPI during a consultation. To decide on a referral, the GPs focus on patient preferences informal sources (e.g. connections with specialists), their own previous experiences, and hospital distance from the patient’s home. Our study confirms various findings from the UK, Denmark, and the Netherlands [24, 35–37].

The GPs feel responsible for coordinating care for their patients, but see no need for using CPI during the referral process. This is partly due to not knowing where to find CPI, but there is also some ignorance regarding the content and outcome expectancy for CPI. This ignorance may influence the ratings they gave. In another study, GPs did not view CPI as a source of information [37]. A precondition for this kind of CPI usage is the reliability of the information and its sources. As in other studies, our GPs reported distrust of the content and validity of CPI [16, 23, 25]. As long as GPs do not trust CPI, other sources of information will remain more important in their referral considerations.

Because CPI makes clear statements on quality differences and providers of high-quality care, it can be seen as a powerful source of information for GPs in selectively choosing a provider as well as in being the patient’s advocate while interpreting and discussing the available data. Regarding selective choice of a provider, a recent Dutch study showed that GP referral patterns were unaffected by report cards, with the exception of outcome indicators for breast cancer [38]. Thus, even if CPI highlights differences in the quality of care, GP referral decisions are not, or are hardly, affected. Consequently, the intended impact of CPI in enabling a selective choice of a provider is not achieved.

Regarding the GP’s role as an advocate, it seems that patients hardly ever introduced CPI. On the basis of our results, we can question whether the GP would use CPI if the patient suggested it. A lack of knowledge and a certain unwillingness both seem to contribute to the GP’s not using this kind of information during the referral process. A recent study [37] suggests an interaction between the GP’s use of CPI and patients’ use of publicly available CPI in the decision-making discussion about referral with their GPs. Hence, if patients were to approach their GPs with publicly available information about quality more often, their GPs would be more likely to have consulted CPI themselves. However, because the patients hardly use CPI, and GPs do not either, the *status quo* continues. A UK study has shown that none of their participating GPs initiated a discussion of differences between services with patients [18]. Approximately half the GPs in our study said that they discussed quality of care differences with their patients. Given their responses, we see that these discussions are not based on publicity available CPI. It may be that patients, despite their not using CPI, may become increasingly critical about differences in quality of care. Ensuring that care quality becomes an issue in the patient consultation can be considered a ‘tipping point’ in the path towards the use of CPI in the referral process.

Despite the GPs’ restraint towards CPI, leaving the choice of provider in the hands of the patient alone worried some GPs as well. In relation to the coordinator role, the GPs in our study feared a further fragmentation of care, as patients might, as a result of CPI, choose providers outside the reach of their professional network. This reasoning has been described in another study as well [39], and it makes sense because it is difficult to predict how and in which cases the benefits of using CPI and the choice of high-quality providers outweigh the threats to continuity of care. New in our study is that GPs link this concern to their own professional role and to the potential weakening of their professional network – they like to keep an overview in their role as the coordinators in the Dutch ‘gatekeeper’ system.

### Implications

Our study has various implications:

- GPs should discuss whether and how to act as supportive agents for their patients using CPI in a way that does justice to their feelings of responsibility, concerns, and practical conditions
- Education of GPs about CPI, its measures, the methodology on which information is based, and the possible better outcomes, as well as teaching them how to discuss CPI with patients
- CPI should be publicized and made available to GPs so that they become aware of the information, can access it easily, and recognize practice variation between hospitals
- Time is required to improve patient engagement in referral discussions (e.g. longer consultations).

### Strengths and limitations

One of this study’s strengths is the use of both qualitative and quantitative data. The GPs interviewed came from innovative and frontrunner general practices. Even though this might have affected the interview results, the participants were drawn from a representative sample of Dutch general practices [40]. We therefore used the survey results to draw a picture of how Dutch GPs use CPI, while the interview results were used mainly to illustrate the quantitative findings. A limitation was the number of CPI-related questions that could be added to the annual LINH survey. Therefore, not every item highlighted in the interviews could be added to the survey in order to quantify our findings. We focussed on the barriers that the GPs encountered without explicitly discussing facilitating factors. The GPs noted their intention to act in the patient’s best interests in the referral considerations, the importance of the free choice of provider for patients, and the discussion of quality of care with patients, though none of these factors included facilitators for the use of CPI.

## Conclusion

General practitioners play a key role in referring patients to hospital care. Their decisions about referrals to hospital care are not based on systematically collected CPI because other referral considerations are more important. CPI is assumed to be an important factor in selective-referral behaviour, as is supporting the patient’s ability to choose a provider of high-quality care by offering more transparency. Despite policy measures that encourage selectively choosing a provider and the expectations that both patients and GPs will make an active and informed choice based on the increasing availability of CPI, both are in a preliminary phase of using this data. Whether and how the GP’s roles in CPI use and patient support should be actively stimulated and supported is still to be determined.

## Abbreviations

CPI: Comparative performance information
GP: General practitioner
M: Mean
s.d.: Standard deviation.
